# Editorial: Omics applications in agriculture systems: unveiling functionality and practicality

**DOI:** 10.3389/fpls.2025.1642175

**Published:** 2025-07-18

**Authors:** Dinesh Adhikary, Joann Mudge, Thiruvarangan Ramaraj

**Affiliations:** ^1^ Agricultural Food and Nutritional Sciences, University of Alberta, Edmonton, AB, Canada; ^2^ Bioinformatics, National Center for Genome Resources, Santa Fe, NM, United States; ^3^ School of Computing, Jarvis College of Computing and Digital Media, DePaul University, Chicago, IL, United States

**Keywords:** OMICS, *Fusarium* head blight, *Ginkgo biloba*, flax, radish, RNA-sequencing

Plants respond to environmental stimuli in a variety of ways. They have developed complex genetic and biochemical networks to adapt to environmental stresses, including biotic, abiotic, herbicide, and metabolic demands (Sharma et al., 2012). With the swift rise of multi-omics technologies, such as genomics, transcriptomics, proteomics, and metabolomics, our ability to characterize molecular mechanisms has led to advances in the understanding of crop resilience, disease resistance, and metabolic regulation (Crandall et al., 2020; Zenda et al., 2021; Adhikary et al., 2024). Researchers in this Research Topic have explored multiple domains across plant sciences, such as the underlying molecular factors related to biotic stress involving *Fusarium* species in wheat and flax (Quintans et al.; Walker et al.). Similarly, the genetic basis of leaf morphology in relict plant species such as *Ginkgo* has been developed (Li et al.). Furthermore, genetic insights into the skin colour variation in radish and strigolactones (SLs) biosynthesis in rice have been explored (Li et al.). Finally, the availability of multi-omics approaches and the utility of harnessing the power of data integration is highlighted in the review article (Sen et al.).

One of the key areas of focus is biotic stress, particularly the threat posed by *Fusarium* head blight (FHB), a devastating fungal disease that affects a majority of cereal crops and leads to significant yield losses and mycotoxin contamination (Shin et al., 2014; Hay et al., 2022; Moonjely et al., 2023). To understand the molecular basis of FHB resistance, Walker et al. performed a comparative transcriptomic analysis of three wheat genotypes: FHB-resistant AC Emerson, FHB-moderately resistant AC Morley, and FHB-susceptible CDC Falcon in response to *F. graminearum*, a dominant causative agent of the disease. The study applied an RNA-sequencing approach and identified key defense mechanisms such as lignin biosynthesis and DON detoxification via UDP-glycosyltransferases, providing insights into the FHB resistance in wheat. Additionally, differential expression of pathogenicity factors in *F. graminearum* was assessed, which offered potential targets for developing resistant wheat varieties.

Moving from biotic stress to hormone-mediated development, another study explored strigolactones (SLs) in rice, a class of carotenoid-derived hormones that regulate plant architecture, response to nutrient availability, and developmental processes, including root and shoot development (López-Ráez et al., 2008; Koltai, 2011; Xu et al., 2019; Li et al., 2023). Li et al. integrated a yeast one-hybridization screening assay and metabolome approach to identify *OsSPL3* as a transcriptional repressor of *OsDWARF10 (OsD10)*, a key gene in SL biosynthesis. The repression of *OsD10* altered the metabolomic profile of polished rice, leading to an increase in amino acids and vitamins. The discovery provided new opportunities to manipulate SL pathways for improving nutritional quality in rice, potentially addressing global food security concerns (Li et al.).

In a related theme of agronomic trait enhancement, pigment accumulation of anthocyanins in radish taproots presents both economic and nutritional value (Khoo et al., 2017). An integrative study using a genome-wide association study and yeast two-hybrid assay has identified a novel genetic locus on the R2 chromosome near *RsMYB1.1* as a key genetic factor regulating skin colour variation in radish, with a large AT-rich insertion in the promoter region of non-red radishes inhibiting anthocyanin biosynthesis (Kim et al.). This presence/absence variation mechanism represents a novel genetic regulation model that could be leveraged for crop improvement through targeted gene editing.

While these studies focus on traits shaped by environmental interactions and selective pressures, evolutionary aspects of plant morphology are also crucial. Since the transition of plants from water to land, terrestrial plants have undergone numerous morphological changes over time. *Ginkgo biloba*, a living fossil, retains ancient characteristics that can provide insights into plant adaptation (Beerling et al., 2001). The unique flabellate (fan-shaped) leaves of *Ginkgo biloba* exhibit distinct anatomical and physiological characteristics compared to other plant leaves. An integrative study involving transcriptomic and metabolomic analyses suggests that endogenous hormones, such as gibberellin (GA), auxin, and jasmonic acid, contribute to leaf shape formation (Li et al.). Additionally, differences in flavonoid and phenolic acid accumulation indicate potential adaptive advantages. Understanding the genetic basis of leaf morphology in relict plant species like *Ginkgo* offers insights into the organ development and the evolution of plants in the terrestrial ecosystem.

Extending the theme of plant resilience, Quintans et al. investigated how beneficial plant-microbe interactions can enhance disease resistance in flax (*Linum usitatissimum* L.). The crop has been challenged by several pests and pathogens (Moyse et al., 2023). *Fusarium* wilt in flax, caused by *F. oxysporum f.* sp. *lini*, is a major agricultural concern. However, research has shown that inoculation with the mutualistic arbuscular mycorrhizal fungus (AMF) *Rhizoglomus irregulare* can mitigate the negative effects of the pathogen (Quintans et al.). This study integrated phenotypic and transcriptomic analysis and investigated the response of flax seedlings to *F. oxysporum* in the presence of AMF *Rhizoglomus irregulare.* The findings revealed that flax prioritizes the expression of mutualism-related genes over conventional defence responses, thereby reducing pathogen-induced growth inhibition. This study highlights the potential of AMF inoculation as a biological control strategy to enhance crop resilience in flax.

Complementing the focus on pathogen resistance, herbicide resistance in weeds presents a significant challenge in modern agriculture (Baucom, 2019). While several studies have explored functional genomics, transcriptomics, proteomics, and metabolomics separately, an integrated approach is needed to fully understand resistance mechanisms, particularly non-target site resistance (Adhikary et al., 2022a, 2022b; Peng et al., 2023; Sen et al., Dong et al., 2024). High-throughput sequencing and molecular profiling can help dissect the complex, multi-pathway responses that enable weeds to survive multiple herbicide modes of action. Especially, multi-omics provides a holistic picture of gene function within complex biological systems. This systems biology approach involving different layers of omics from molecular and cellular levels enhances our ability to precisely identify biomarkers that are related to various agronomic traits, including herbicide resistance, and develop more effective weed management strategies. Through this approach, we can link genes to phenotypes, capture regulatory mechanisms, identify post-translational or post-transcriptional modifications, which potentially affect gene functions, most importantly, it improves the accuracy of gene annotation and discovery. By continuing to explore these molecular pathways, we can accelerate the development of crops with enhanced resistance, improved nutrition, and greater adaptability to environmental challenges, ultimately paving the way for a more sustainable and food-secure future.

These studies collectively emphasize the transformative potential of multi-omics approaches in revealing the genetic and molecular mechanisms underlying key plant traits, evolutionary adaptations, and resistance strategies. Future research should prioritize the integration of various omics technologies, such as genomics, transcriptomics, proteomics, and metabolomics, to develop a holistic model of plant stress response. In parallel, functional validation strategies such as genome editing, gene overexpression, and gene silencing are essential to move beyond descriptive omics data and rigorously confirm the roles of candidate genes. This approach not only enhances the scientific value of research outputs but also addresses a critical gap in basic science, where functional characterization remains limited. Furthermore, validating gene function strengthens the biological relevance of findings and lays the groundwork for translational applications in crop improvement. Additionally, leveraging beneficial plant-microbe interactions can contribute to the development of sustainable and resilient agricultural systems.
